# Organ system-involvement in SLE and relationship with demographic factors, disease duration and health-related quality of life in childhood SLE

**DOI:** 10.1186/1546-0096-10-S1-A22

**Published:** 2012-07-13

**Authors:** Lakshmi N Moorthy, Maria J Baratelli, Margaret GE Peterson, Afton L Hassett, Alexa B Adams, Laura V Barinstein, Emma J MacDermott, Elizabeth C Chalom, Karen Onel, Linda I Ray, Jorge Lopez-Benitez, Christina Pelajo, Kathleen A Haines, Daniel J Kingsbury, Victoria W Cartwright, Philip J Hashkes, Nora G Singer, Gina A Montealegres, Ingrid Tomanova-Soltys, Andreas O Reiff, Sandy D Hong, Thomas JA Lehman

**Affiliations:** 1 Childrens Hospital LA, Los Angeles, CA, USA; 2 Hackensack University Medical Center, Hackensack, NJ, USA; 3 Hospital for Special Surgery, New York, NY, USA; 4 Legacy Emanuel Children's Hospital, Portland, OR, USA; 5 Maimonides Medical Center, Brooklyn, NY, USA; 6 MetroHealth Medical Center, Cleveland, OH, USA; 7 Shaare Zedek Medical Center, Jerusalem, Israel, USA; 8 St. Barnabas Medical Center, New Brunswick, NJ, USA; 9 Tufts Medical Center, Boston, MA, USA; 10 UMDNJ-Robert Wood Johnson Medical School, New Brunswick, NJ, USA; 11 University Hospitals Case Medical Center, Cleveland, OH, USA; 12 University of Chicago, Chicago, IL, USA; 13 University of Iowa Children's Hospital, Iowa City, IA, USA; 14 University of Medicine and Dentistry of NJ (UMDNJ)- Robert Wood Johnson Medical School, New Brunswick, NJ, USA; 15 University of Michigan Medical School, Ann Arbor, MI, USA; 16 University of Mississippi Medical Center, Jackson, MS, USA

## Purpose

Damage in childhood systemic lupus erythematosus (SLE) affects ocular, musculoskeletal, neuropsychiatric, renal, cardiovascular, peripheral vascular, and skin domains and damage can affect their health related quality of life (HRQOL). Aggressive treatments have improved survival in childhood SLE, but the disease is still associated with significant morbidity. The objective of our multicenter study is to examine the organ system-involvement in childhood SLE and the relationship of damage with HRQOL, age, gender, ethnicity and disease duration.

## Methods

In this cross-sectional study, children ≤18 years with SLE and parents completed the Simple Measure of the Impact of Lupus Erythematosus in Youngsters© (SMILEY©) and physicians measured Systemic Lupus International Collaborating Clinics/ACR Damage Index (SDI). SMILEY© is a new, brief, 24-item HRQOL assessment tool for pediatric SLE, that has recently been validated in US English. The four domains are: Effect on self, Limitations, Social and Burden of SLE. Responses are in the form of a 5-faces scale for easy comprehension. Higher percentage scores indicate better HRQOL. Contingent upon the data distribution of the above variables, we used student t-test, Mann-Whitney U test or the Kruskal-Wallis (KW) test to examine the relationship of SDI with age, gender, ethnicity, disease duration and HRQOL.

## Results

Out of a total of 169 children (17% male), 59 children (35%) had any damage (SDI score>0). Their age, damage and disease duration and specific organ-system involvement is given in table 1. Their ethnicities were: Black (39%), Asian or Pacific Islander (12%), Latino (27%), White (18%) and other (5%). Children predominantly had renal, neuropsychiatric, skin, and musculoskeletal involvement. Significant difference was found in damage with disease duration (p=0.005, Mann Whitney U test). There was no significant difference in damage in patients with different gender, ages or ethnicity. Parent SMILEY© total and all domain scores were decreased in patients with damage compared to patients without damage (table 2), but only the Effect on self domain scores was statistically significant (p=0.02). The child SMILEY© total, Limitation and Effect on self domain scores were lower in patients who had any damage.

**Table 1:** SDI scores and organ-system involvement, and HRQOL scores

Variables	Descriptives (n=169)
**SDI total score**	

Mean ± SD (range)(n)	36±39 (1-183)
Median	Median =24

**Age (years)**	15 ± 3 (6-18)

**Disease duration (months)**	

Mean ± SD (range) (n)	36±39 (1-183)
Median	Median =24

**Renal** %(n)	17 (29)

**Neuropsychiatric** %(n)	14 (24)

**Skin** %(n)	11 (18)

**Musculoskeletal** %(n)	6 (10)

**Peripheral vascular** %(n)	3 (5)

**Ocular** %(n)	2 (4)

**Pumonary** %(n)	2 (4)

**Diabetes** %(n)	2 (3)

**Cardiovascular** %(n)	2 (3)

**Gastrointestinal** %(n)	1(2)

**Premature gonadal failure** %(n)	1 (2)

**Malginancy** %(n)	0 (0)

**SMILEY total score**	

Mean ± SD (range) (n) Child	65 ± 14 (33-98) (166)
Parent	62 ± 15 (28-96) (153)

**Table 2:** Means of parent total and domain scores of SMILEY for patients with no damage (SDI=0) and patients with any damage (SDI>0)

Parent SMILEY scores	SDI score=0 (n)	SDI score >0 (n)
Effect on self domain	63±18 (96)	57±16 (55)
Limitation domain	62±19 (97)	59±16 (55)
Social domain	78±18 (97)	74±17 (55)
Burden of SLE domain	57±16 (96)	54±17 (55)
Total score	64±15 (97)	60±14 (55)

## Conclusion

Damage in childhood SLE is significantly related to disease duration. Renal, neuropsychiatric, skin, and musculoskeletal systems are predominantly involved in our US cohort, which is similar to previous studies. The impact of damage on children’s HRQOL as perceived by parents may be different from the children’s perception and needs further examination.

## Disclosure

Lakshmi N. Moorthy: Arthritis Foundation, 2; Maria J. Baratelli: Arthritis Foundation, 2; Margaret G.E. Peterson: Arthritis Foundation, 2; Afton L. Hassett: Arthritis Foundation, 2; Alexa B. Adams: None; Laura V. Barinstein: None; Emma J. MacDermott: None; Elizabeth C. Chalom: None; Karen Onel: None; Linda I. Ray: None; Jorge Lopez-Benitez: None; Christina Pelajo: None; Kathleen A. Haines: None; Daniel J. Kingsbury: None; Victoria W. Cartwright: None; Philip J. Hashkes: None; Nora G. Singer: None; Gina A. Montealegres: None; Ingrid Tomanova-Soltys: None; Andreas O. Reiff: None; Sandy D. Hong: None; Thomas J. A. Lehman: None.

